# TPCpp-10M: Simulated proton-proton collisions in a time projection chamber for AI foundation models

**DOI:** 10.1016/j.dib.2025.112393

**Published:** 2025-12-16

**Authors:** Shuhang Li, Yi Huang, David Park, Xihaier Luo, Haiwang Yu, Yeonju Go, Christopher Pinkenburg, Yuewei Lin, Shinjae Yoo, Joseph Osborn, Christof Roland, Jin Huang, Yihui Ren

**Affiliations:** a Department of Physics, Columbia University, New York, NY, USA; b AI Department, Brookhaven National Laboratory, Upton, NY, USA; c Nuclear and Particle Physics Department, Brookhaven National Laboratory, Upton, NY, USA; d Department of Physics, Massachusetts Institute of Technology, Cambridge, MA, USA

**Keywords:** RHIC, Charged-particle tracking, Monte Carlo, Geant4, Pythia, Spacepoints, Machine learning, Benchmarking

## Abstract

Scientific foundation models hold great promise for advancing nuclear and particle physics by improving analysis precision and accelerating discovery. Yet, progress in this field is often limited by the lack of openly available large-scale datasets, as well as standardized evaluation tasks and metrics. Furthermore, the specialized knowledge and software typically required to process particle physics data pose significant barriers to interdisciplinary collaboration with the broader machine learning community. This work introduces a large, openly accessible dataset of 10 million simulated protonproton collisions, designed to support self-supervised training of foundation models. To facilitate ease of use, the dataset is provided in a common NumPy format. In addition, it includes 70,000 labeled examples spanning three well-defined downstream tasks – track finding, particle identification, and noise tagging – to enable systematic evaluation of the foundation model’s adaptability. The simulated data are generated using the Pythia Monte Carlo event generator at a center-of-mass energy of $\sqrt{s}=200\;GeV$ and processed with Geant4 to include realistic detector conditions and signal emulation in the sPHENIX Time Projection Chamber at the Relativistic Heavy Ion Collider, located at Brookhaven National Laboratory. This dataset resource establishes a common ground for interdisciplinary research, enabling machine learning scientists and physicists alike to explore scaling behaviors, assess transferability, and accelerate progress toward foundation models in nuclear and high-energy physics. The complete simulation and reconstruction chain is reproducible with the sPHENIX software stack. All data and code locations are provided under Data Accessibility.

Specifications Table

**Table:** 

Subject	Physics
Specific subject area	Charged-particle tracking simulation in a time projection chamber (TPC) at Relativistic Heavy Ion Collider (RHIC)
Type of data & formats	NumPy compressed archive
Data collection	proton+proton (p+p) collision events generated with Pythia 8.307 (Detroit tune) [1], [2] at center-of-mass energy $\sqrt{s}=200\;GeV$; simulated with Geant4[3] (FTFP_BERT_HP) via the as-built sPHENIX detector and 1.4T magnetic field; TPC signals digitized and reconstructed into spacepoints using sPHENIX software. Labeled set includes spacepoint-to-truth associations;
Data source location	Brookhaven National Laboratory, Upton, NY, USA (simulated under sPHENIX software environment).
Data accessibility	Repository name: **Zenodo**
	Data identification number (DOI): 10.5281/zenodo.16970029 Direct URL to data: https://doi.org/10.5281/zenodo.16970029 Instructions for access: Public, anonymous download; dataset provided as compressed archives; example loaders and documentation included in the repository.
Related research article	None

## 1 Value of the Data

• **Supports foundation model training.** The dataset provides 10000000 unlabeled events suitable for large-scale self-supervised or contrastive pretraining, complemented by 70000 labeled events for fine-tuning. This enables scalable applications of machine learning to charged-particle tracking.
• **Fills a gap in open high-energy physics datasets.** Most publicly available tracking datasets are based on silicon detectors (e.g., strip or pixel trackers) in high-energy particle collisions. In contrast, this dataset is built on a large, gas-filled TPC, the central tracker in sPHENIX and many other nuclear physics experiments. By providing realistic TPC spacepoints with truth associations, it enables research on tracking challenges unique to gaseous detectors, such as long drift distances, diffusion, and complex pattern recognition across several tens of layers, compared to the ∼10 precise two-dimensional layers typically found in silicon trackers.
• **Benchmarking resource.** The labeled subset includes ground-truth information for three standard downstream tasks – track finding, particle identification, and noise tagging – along with recommended metrics, establishing a common benchmark for fair and reproducible evaluation.

## 2 Background

**Fig. 1:**
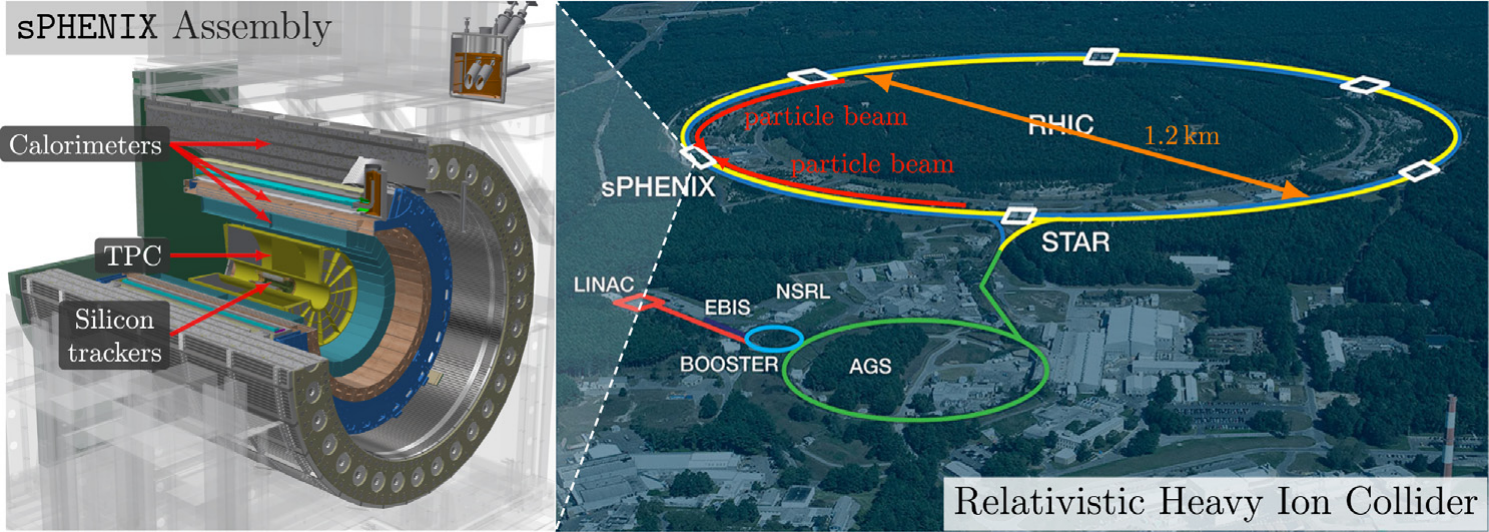
RHIC and sPHENIX experiment (context figure) [4].

**Fig. 2:**
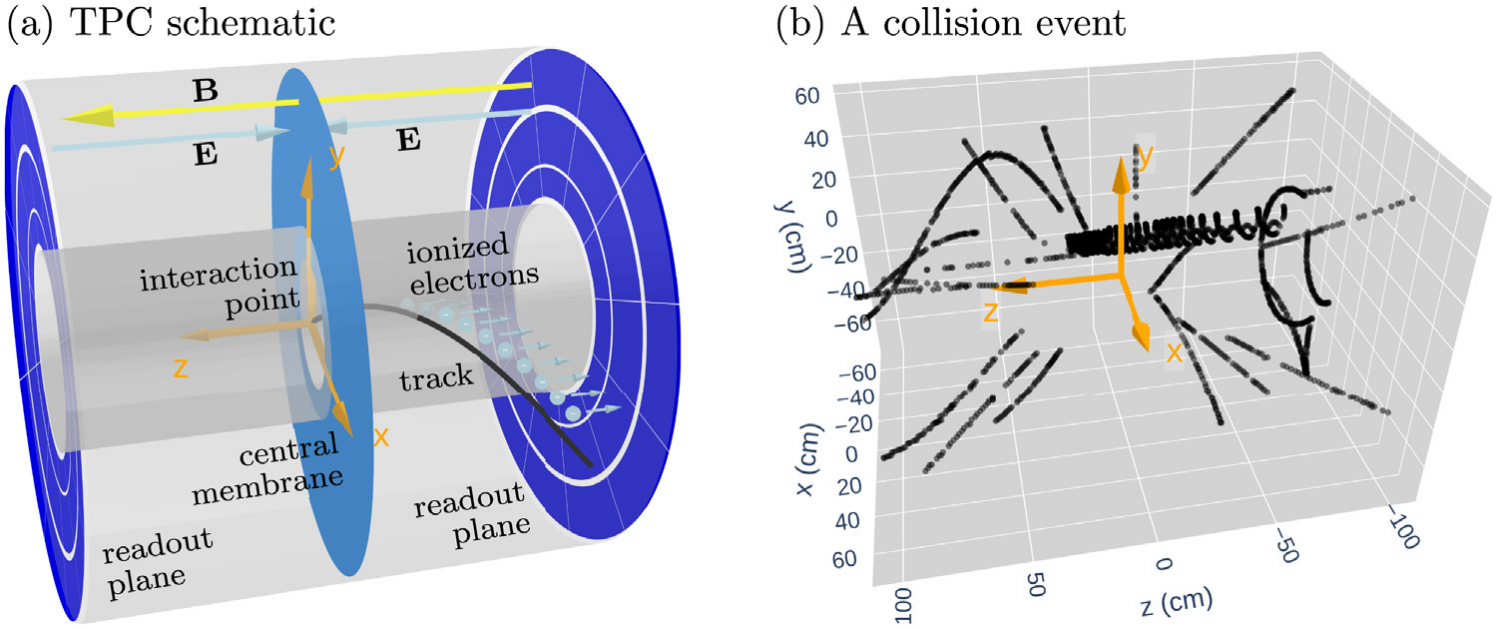
TPC schematic and collision event. Panel (a): The TPC schematic, where proton beams travel along the z direction. The electric field E drives ionization electrons to the readout planes, while the magnetic field B bends charged particle trajectories. Panel (b): An event display of reconstructed spacepoints in the TPC.

In nuclear and particle physics experiments, tracking detectors are used to measure and reconstruct the trajectories of charged particles. These measurements are crucial for determining momenta, identifying particle species, and studying underlying physics processes. The TPC provides three-dimensional (3D) spacepoints that reconstruct the trajectories of particles traversing the detector. Fig. 1 shows the sPHENIX TPC at RHIC is a cylindrical gas-filled detector with an instrumented region featuring an inner radius of ∼32cm, outer radius of ∼78cm, and length of ∼211cm in a 1.4T magnetic field [5]. The TPC records the ionization signals produced as charged particles pass through the gas volume. Fig. 2 illustrates how the ionized electrons drift under a uniform electric field produced by the central membrane toward the readout planes, where their arrival times encode the longitudinal (z) coordinate and the channel locations in the readout planes provide the transverse (rϕ) coordinates. In this way, each charged particle leaves a series of high-resolution spacepoints (∼150μm in rϕ; ∼750μm in z) along its trajectory. As the central component of tracking in sPHENIX, the TPC provides precise momentum measurements from particle trajectories and particle identification.

This dataset was compiled to provide a public, high-fidelity resource of simulated minimum-bias p+p collisions at $\sqrt{s}=200\;GeV$, motivated by the need for openly accessible data to develop and benchmark machine learning and reconstruction algorithms for TPC tracking. Such collisions, standard at RHIC, offer a controlled environment for testing methods before tackling complex heavy-ion collision data. It is important to note that the TPC occupancy in simulated protonproton events (typically ranging from a few hundred to several thousand spacepoints per event) is significantly lower than in heavy-ion collisions. On average, heavy-ion collisions exhibit occupancies about one order of magnitude higher, and in extreme high-multiplicity events they can reach two orders of magnitude higher. This dataset ensures portability and reproducibility with clearly defined units and truth associations, fostering method development across physics and data science communities without requiring proprietary software access. The large unlabeled set (10M events) is particularly well suited for training foundation models via self-supervised learning, addressing the need for scalable, high-fidelity datasets in charged-particle tracking.

## 3 Data Description

**Fig. 3:**
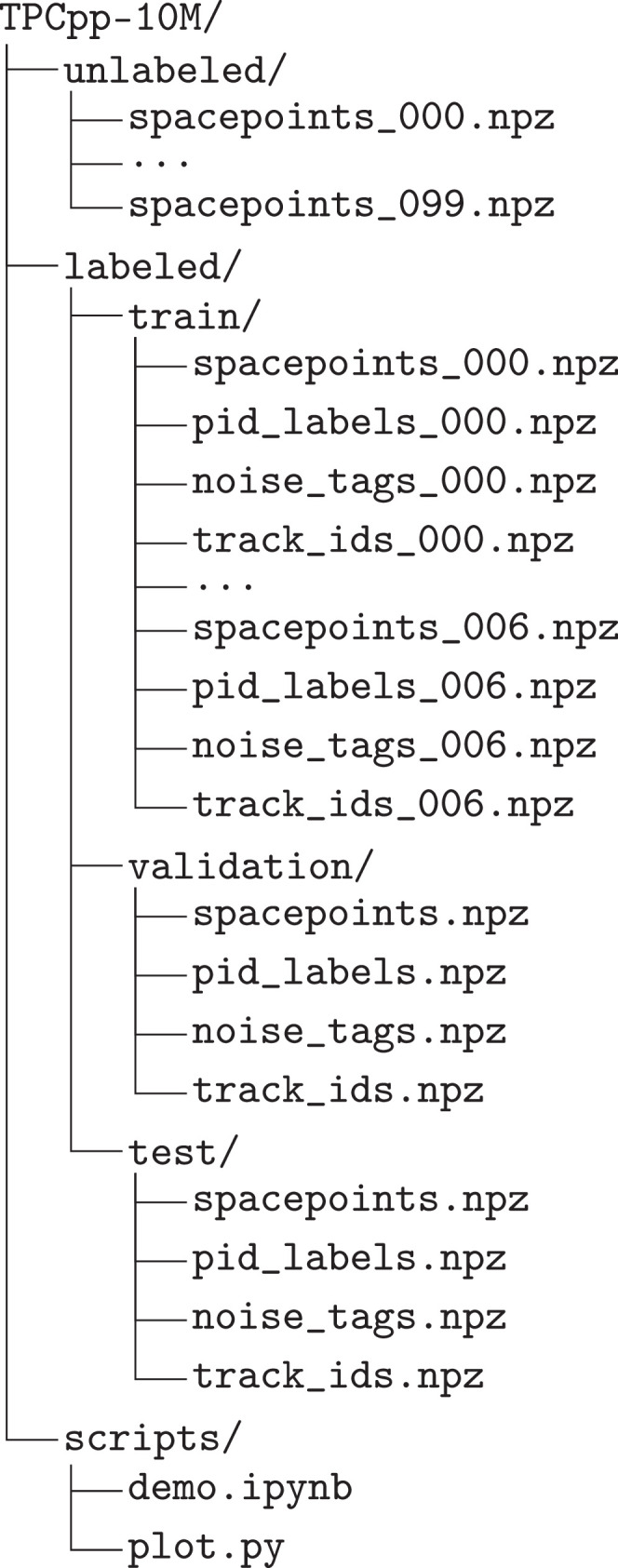
Folder structure of the TPCpp-10M dataset.

The dataset repository contains simulated p+p collision data at $\sqrt{s}=200\;GeV$ for the sPHENIX TPC, organized into three top-level folders. All data files are stored in .npz format, which can be read with Python libraries, such as NumPy. The TPC is a cylindrical detector with its z-axis along the beam direction, centered at the detector origin. Spacepoint coordinates (x,y,z) are reported in Cartesian coordinates (cm). Fig. 3 provides a summary of the folder structure.

• unlabeled/: Contains 10M minimum-bias events split into 100 shards (named spacepoints_[000-099].npz), each with 100000 events. Each file stores:
  • spacepoints: Array of shape $\left(N_{i},4\right)$ with columns (E,x,y,z), where x,y,z are positions (cm) in the TPC active volume and E is the ionization signal (ADC units).
• labeled/: Contains overall 90k events with full truth information, organized into three subsets:
  • train/: 7 shards (*_000.npz to *_006.npz), each with 10k events.
  • validation/: A single file per array type for 13k events.
  • test/: A single file per array type for 7k events.
  Each labeled subset includes four aligned arrays:
  • spacepoints: Array $\left(N_{i},4\right)$ with (E,x,y,z).
  • track_ids: Integer array $\left(N_{i}\right)$ that assigns each spacepoint to a unique truth track identifier.
  • noise_tags: Integer array $\left(N_{i}\right)$ with one entry per spacepoint. For each spacepoint, $N_{i}=1$ if the matched tracks transverse momentum $p_{T}<60\;MeV/c$ and 0 otherwise. This flags spacepoints produced by particles that are unlikely to reach the TPC active volume.
  • pid_labels: String array $\left(N_{i}\right)$ that is derived from Particle Data Group [6] codes and grouped into five categories (labels): *charged pion* (1), *charged kaon* (2), *proton/antiproton* (3), *electron/positron* (4), and *others* (0).
• scripts/: Contains Python utilities for data handling:
  • demo.ipynb: Example code for data loading and visualization.
  • plot.py: Helper functions for data visualization.

**Fig. 4:**
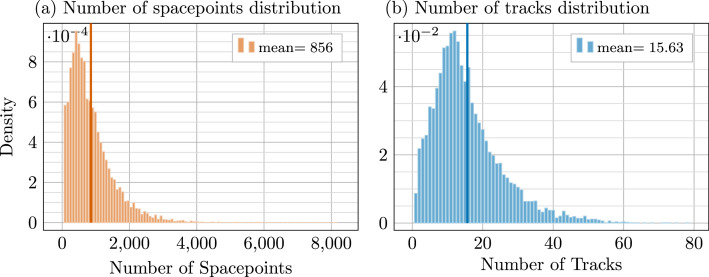
Panel (a): Distribution of spacepoints per event. Panel (b): distribution of truth tracks per event.

Each event includes reconstructed TPC spacepoints, as well as (for the labeled set) truth associations and per-track kinematics. As shown in Fig. 4, event complexity ranges from a few hundred to several thousand spacepoints, with truth tracks spanning from fewer than 10 to nearly 100 per event.

Given the information in the labeled dataset, several reconstruction tasks can be posed. The following defines three tasks: track finding, noise tagging, and particle identification (PID), along with the corresponding labels derivable from the provided truth.

**Track finding:** assign every reconstructed spacepoint to a track identity using the provided truth associations. The label for each spacepoint is the integer track_id of its matched truth track. *Note*: Evaluation may be restricted to primaries or tracks passing kinematic cuts, but the labels themselves are unchanged.
**Noise tagging:** binary classification noting whether or not a spacepoint is noise.
**PID:** multiclass classification of truth tracks into the five most commonly seen chargeconjugate categories: $\pi^{\pm }$ (charged pion), $K^{\pm }$ (charged Kaon), $p/\bar{p}$ (proton/antiproton), $e^{\pm }$ (electron/positron), and all other particles.

**Fig. 5:**
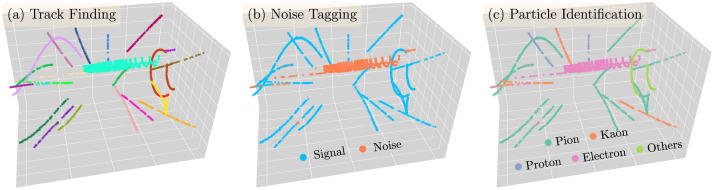
Downstream tasks ground truth labels.

**Fig. 6:**
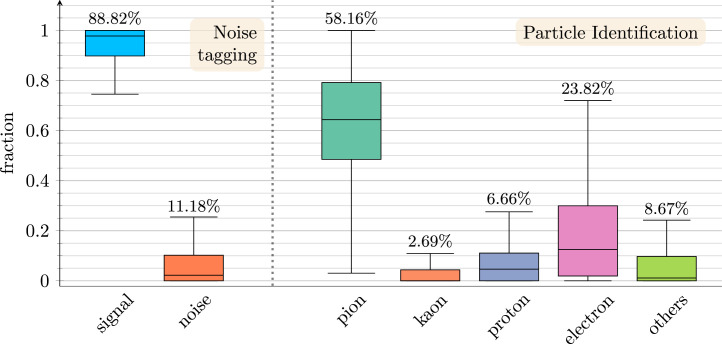
Box plots of per-event ground truth class ratios in the test subset of the labeled dataset, shown for noise tagging and particle identification tasks. The horizontal line within each box denotes the median per-event class ratio, the box edges indicate the interquartile range, and the whiskers show the full range across events. The dataset-wide average fractions are annotated above the whiskers.

Fig. 5 shows an example event display with all downstream labels. The labels are constructed directly from the provided truth associations and per-track kinematics. Class ratios for the noise and PID tasks in the labeled subset are shown in Fig. 6.

### Evaluation metrics

The dataset includes recommended evaluation metrics for the supported machine learning tasks. For noise tagging and PID, standard classification metrics (accuracy, precision, and recall) are recommended.

For track finding, double-majority matching [7] is suggested to assess efficiency and purity, following the terminologies and conventions proposed in [7] and [8].

Given a predicted track candidate labeled by t and a ground truth particle with label p, we define:

• c(t,p) as the number of hits that have predicted label t and true label p
• c(:,p) as the number hits with true label p
• c(t,:) as the number of hits with predicted label t

and

• **Hit efficiency** as c(t,p)/c(:,p)
• **Hit purity** as c(t,p)/c(t,:).

To match a track candidate to a particle, we follow the **double-majority rule** wherein the track candidate t is matched to particle p if – and only if – both hit efficiency and purity exceed 50%. This stringent rule guarantees that a track candidate can be matched to at most one particle, and a particle can also be matched by no more than one track candidate.

After the matching is done, the metrics that evaluate the overall tracking performance can be defined. Let T be the number of track candidates, P be the number of ground truth particles, and M be the number of matches. The efficiency and purity of tracking can be defined as:

• **Tracking efficiency** as M/P
• **Tracking purity** as M/T.

## 4 Experimental Design, Materials, and Methods

**Event generation and transport.**Particles in minimum-bias p+p collision events at $\sqrt{s}=200\;GeV$ are generated with Pythia 8.307 [1] (Detroit tune [2]). The particles are transported with Geant4 [3] using the FTFP_BERT_HP physics list via the as-built sPHENIX geometry and the measured 1.4 T solenoidal field. The simulation includes continuous energy loss, multiple scattering, secondary particle production, and decay processes when interacting with the detector material.**TPC response, digitization, and reconstruction.**Ionization signals are simulated and digitized with realistic channel-dependent gain and noise, including shaping and zero suppression. The resulting digitized 3D TPC hits are subsequently clustered into spacepoints using sPHENIX clustering algorithms. The spacepoints serve as the fundamental units for pretraining and downstream tasks, such as track finding and PID.**Data preprocessing.**All spacepoints belonging to tracks that contain fewer than five spacepoints are removed because they are too short to represent a physically meaningful particle trajectory in the TPC. Such short segments almost always originate from detector noise or random hit combinations rather than from true charged particles. In addition, diffractive collision processes, similar to elastic scattering, produce only a small number of particles near the beam direction and result in little activity in the central region covered by the TPC, making them uninformative for assessing central tracking performance. After removing short tracks, the number of remaining spacepoints are counted, and the event is rejected if it contains fewer than 20 spacepoints.**Code and reproducibility.**All of the code used to drive the Pythia event generator and Geant4 detector simulations, as well as the downstream emulation and reconstruction chain (digitization; spacepoint reconstruction), is sourced from the sPHENIX software stackcore simulation/reconstruction libraries and supporting infrastructure: [9], [10], [11], [12]. For exact reproducibility, we provide repository URLs and the commit hashes used to generate this release, as well as the corresponding sPHENIX analysis build release number (ana.435) from the CernVM File System (CVMFS) [13], which encapsulates the full build environment.

## Limitations

The dataset is simulation-based and reflects the detector, material, and electronics models used. While the overall detector response is realistic, certain effects present in real data are not explicitly modeled in this release, such as space-charge distortions, time-dependent gain variations, and imperfect channel calibrations. These effects can influence local hit positions and energy deposits at the few-percent level in data. Consequently, while the dataset is representative of realistic detector conditions, quantitative differences from real data are expected for such low-level observables.

## Ethics Statement

The authors confirm this work does not involve human subjects, animal experiments, or data collected from social media platforms and follows the ethical requirements for publication found in *Data in Brief*.

## CRediT authorship contribution statement

**Shuhang Li:** Conceptualization, Methodology, Data curation, Writing – original draft. **Yi Huang:** Software, Validation, Writing – original draft, Visualization. **David Park:** Conceptualization, Writing – review & editing. **Xihaier Luo:** Conceptualization, Writing – review & editing. **Haiwang Yu:** Validation, Writing – review & editing. **Yeonju Go:** Validation, Writing – review & editing. **Christopher Pinkenburg:** Resources, Software, Writing – review & editing. **Yuewei Lin:** Conceptualization, Writing – review & editing. **Shinjae Yoo:** Writing – review & editing. **Joseph Osborn:** Software, Writing – review & editing. **Christof Roland:** Software, Writing – review & editing. **Jin Huang:** Funding acquisition, Supervision, Conceptualization, Writing – review & editing. **Yihui Ren:** Funding acquisition, Project administration, Supervision, Conceptualization, Writing – review & editing.

## Data Availability

ZenodoTPCpp-10M: Simulated proton-proton collisions in Time Projection Chamber for AI Foundation Models (Original data)

## References

[1] Sjöstrand T., Ask S., Christiansen J.R., Corke R., Desai N., Ilten P., Mrenna S., Prestel S., Rasmussen C.O., Skands P.Z. (2015). An introduction to PYTHIA 8.2. Comput. Phys. Commun..

[2] Aguilar M.R., Chang Z., Elayavalli R.K., Fatemi R., He Y., Ji Y., Kalinkin D., Kelsey M., Mooney I., Verkest V. (2022). pythia8 underlying event tune for RHIC energies. Phys. Rev. D.

[3] Agostinelli S. (2003). GEANT4 - A Simulation Toolkit. Nucl. Instrum. Meth. A.

[4] Brookhaven National Laboratory, sPHENIX Detector at RHIC, 2025, (https://www.bnl.gov/rhic/sphenix.php). Accessed: 2025-07-19.

[5] Klest H. (2020). Overview and design of the sPHENIX TPC. J. Phys. Conf. Ser..

[6] Particle Data Group (2024). Review of particle physics. Phys. Rev. D.

[7] Ju X., Murnane D., Calafiura P., Choma N., Conlon S., Farrell S., Xu Y., Spiropulu M., Vlimant J.-R., Aurisano A. (2021). Performance of a geometric deep learning pipeline for HL-LHC particle tracking. Eur. Phys. J. C.

[8] Garcia D., Selvaggi M., Francois B. (2025). Geometric graph neural network based track finding. openreview.

[9] sPHENIX Collaboration, coresoftware: Core simulation and reconstruction software for the sPHENIX experiment, tagged at b849eba5c2cf8ada510d036aa9b9499cb31f0513, 2025a, (https://github.com/sPHENIX-Collaboration/coresoftware/tree/b849eba5c2cf8ada510d036aa9b9499cb31f0513, Accessed August 3, 2025; used in the official sPHENIX build, including downstream emulation and reconstruction.

[10] sPHENIX Collaboration, acts: A common tracking software toolkit for sPHENIX, tagged at 33fc284f238a24405bcd6c2de3260f370d6f8403, 2025b, (https://github.com/sPHENIX-Collaboration/acts/tree/33fc284f238a24405bcd6c2de3260f370d6f8403, Accessed August 3, 2025; part of the official sPHENIX build environment.

[11] sPHENIX Collaboration, macros: Analysis and utility macros for sPHENIX, tagged at 661f781db23352a3fa72055ce7dbf1a0ee1c2167, 2025c, (https://github.com/sPHENIX-Collaboration/macros/tree/661f781db23352a3fa72055ce7dbf1a0ee1c2167, Accessed August 3, 2025; part of the official sPHENIX build environment.

[12] sPHENIX Collaboration, calibrations: Calibration code for sPHENIX, tagged at a3e66e69635514813ee3e20bf18b2bd59787b503, 2025d, (https://github.com/sPHENIX-Collaboration/calibrations/tree/a3e66e69635514813ee3e20bf18b2bd59787b503, Accessed August 3, 2025; part of the official sPHENIX build environment.

[13] Blomer J., Buncic P., Fuhrmann T. (2011). International Conference for High Performance Computing, Networking, Storage and Analysis.

